# Comment on “A Randomized Trial of Cardiac Catheterization With Fasting Versus Liberal Oral Intake: The CALORI Trial”

**DOI:** 10.1016/j.jscai.2025.102628

**Published:** 2025-03-11

**Authors:** Ibrahim Nagmeldin Hassan, Mohamed Ibrahim

**Affiliations:** University of Khartoum, Faculty of Medicine, Khartoum, Sudan; Al Neelain University Faculty of Medicine, Khartoum, Sudan

The CALORI Trial provides valuable insights into the impact of liberal oral intake strategies on patient well-being before cardiac catheterization.^1^ While its conclusions are promising, several methodological limitations warrant discussion.

First, the single-center design significantly limits the generalizability of the findings. Patient demographics, institutional practices, and procedural expertise vary widely across regions, particularly in multiethnic or resource-limited settings. Conducting multicenter trials would provide more robust evidence to support the universal implementation of these recommendations.

Second, the trial permitted “near complete autonomy” in the type and quantity of oral intake for nonfasting patients. While this reflects real-world settings, the absence of clear guidelines introduces variability that may confound the results. Future studies should consider specifying standardized intake protocols to ensure consistency across participants.

Additionally, the trial excluded high-risk groups, such as those with severe gastroesophageal reflux or those undergoing complex interventions. While this exclusion is justifiable from a safety perspective, it limits the applicability of the findings to these vulnerable populations. The observed lack of adverse events in the trial cannot be extrapolated to settings with higher-risk patients.

Lastly, the study’s primary focus on patient-reported outcomes, though important, should be complemented by objective measures, such as procedure duration or clinical efficiency. While patient comfort is essential, ensuring no trade-offs in procedural safety and efficacy is equally critical.

In conclusion, the CALORI Trial raises important questions about the necessity of prolonged fasting before cardiac catheterization. However, broader investigations that use standardized protocols and diverse populations are required to translate these findings into practice in a reliable manner.
